# ‘It Isn't Charity because We've Paid into it’: Social Citizenship and the Moral Economy of Welfare Recipients in the Wake of 2012 UK Welfare Reform Act

**DOI:** 10.1007/s11133-021-09505-z

**Published:** 2022-02-10

**Authors:** Darren Thiel

**Affiliations:** Department of Sociology, University of Essex, Wivenhoe Park, Colchester, CO4 3SQ UK

**Keywords:** Austerity, Value, Poverty, Rights, Exclusion, Boundaries, Bedroom tax, Benefit cap

## Abstract

Drawing on interviews with welfare claimants living in Essex, UK, this article examines the material and symbolic effects of the UK government’s 2012 Welfare Reform Act, and it highlights the participants’ interpretations of and responses to that. In reaction to their sense of material and symbolic exclusion, participants made moral claims for their inclusion through a notion of social citizenship based on collective reciprocity and care. They claimed to have paid-in to the national purse in various material and moral ways until circumstances outside of their control meant they could no longer do so. They thus asserted a moral-economic right to social inclusion and an ensuing right to receive adequate, non-stigmatised, and non-punitive welfare. These moral-economic claims differ from other, more public, counter-narratives to welfare reform and government austerity, and they assert a clear but subtle opposition to the market-bound logic of the reform*.*

## Introduction: Welfare Reform, Austerity, and the Feckless Underclass

In the wake of the 2008 financial crisis, a programme of fiscal austerity was introduced by the Conservative-led UK coalition government. As part of this, the 2012 Welfare Reform Act was launched in order to reduce rising welfare costs through cuts to welfare payments and ostensibly by increasing the numbers of people in paid employment:

We are creating a [welfare] system based on *fairness*: providing *value* for money and placing greater emphasis on personal *responsibility*. The Reforms will ensure that the system is *fair* to the British taxpayer and people in *genuine* need of support. (DWP 2014, 3. Emphasis added)

Although, later, in 2018, the UK government announced the ‘end of fiscal austerity’ (BBC 2018) and, during the COVID 19 pandemic, provided generous levity for furloughed workers and businesses, this did not conclude welfare austerity and the 2012 Reform Act largely continues its implementation.^1^

The Act combined early changes to welfare provision, described below, with the highly publicised Universal Credit system that will further reorganise and ‘reform’ benefit payments (see DWP 2015). This paper charts the early effects of welfare reform on welfare claimants in Essex before the introduction of the Universal Credit system, which, if early reports are accurate (for example, Brewer et al. 2019; Trussell Trust 2018), is likely to only amplify many of the stark moral and financial issues that are raised in this article.

Not dissimilar to elements of the US 1996 Welfare reform Act (see Hays 2003), the UK Act introduced increased conditionality for welfare claims, ramping up behavioural and financial tests for welfare entitlements, and it instigated a number of financial penalties in order to encourage behavioural change. For instance, new, stricter, actuarial assessments (‘Work Capability Assessments’) were initiated to test the extent of the disability of those claiming not to be able to do paid work due to ill-health. The Act also introduced a number of financial reductions for welfare claimants including: a cap on the upper limit paid out for benefits (the ‘Benefit Cap’); financial penalties for ‘over-occupying’ social housing (the Over Occupation Penalty – or ‘bedroom tax’); and obligatory contributions towards council tax from which welfare recipients were previously exempt (see Table 1).

**Table 1:** Welfare reform changes and penalties

The Benefit Cap	A limit to the upper levels of benefits paid out – including those for housing. Rates were capped at £350 (approximately $525) per week for single adults with no children; £500 ($750) per week for single parents living with children; and £500 ($750) for couples with or without children. The cap particularly affects people in the south of England where rents are high
Council Tax benefits deductions	Welfare claimants required to pay around 10% towards their yearly council tax bills. Council tax is a flat-rate tax levied at households and is unrelated to incomes. It cost an approximate average of £1450 ($2,175) in 2014
Over-Occupation Penalty (OOP)	Also known as the Spare Room Subsidy or ‘Bedroom tax.’ Involves a deduction in housing benefit payments when residents are deemed to under-occupy a property (i.e., when they have a spare room). Involves 14% deduction for one spare room, and 25% for two or more
Discretionary Housing Allowance	Temporary grants (for between three and six months) awarded by local authorities to pay for OOP deductions when tenants can prove they either need the spare room or cannot reasonably move to a smaller dwelling
Personal Independent Payment (PIP)	Introduced to replace the previous main benefit for people with disabilities – the Disability Living Allowance. It involves stricter and more regular assessments, and more employment training programmes

Surrounding reform, government statements pertaining to fairness, responsibility, and genuine need had also revitalised an enduring moral discourse that paints welfare claimants as members of an irresponsible underclass who are not really in need of support but who, rather, *choose* a life on welfare and then become dependent on it (Jensen and Tyler 2015; Slater 2012; Wiggan 2012). This image, and much questionable government-linked research to support it, has been refuted by numerous studies (Dodson 1998; Hayes 2003; Hills 2015; MacDonald and Marsh 2005; MacDonald et al. 2014; Shildrick et al. 2012). Yet, despite this, government and media messages continue to find extreme examples of welfare claimants and parade them as general – providing moral legitimation to the stigmatising and punitive nature of welfare reform (Boland and Griffin 2016; Jensen and Tyler 2015). Combined with a market-based logic about austerity and affordability, this dominant morality has stifled effective critique and reduced the power of any alternative narratives to austere welfare (Clarke and Newman 2012; Morris 2016).

Based on interviews with 40 social housing tenants living in Essex receiving various welfare provisions, this article identifies the dominant narratives that participants formed in relation to the difficult financial situations and increased stigma that they had been placed under by the reform. While some of the findings chime with those of other studies in identifying how welfare recipients passed stigma away from themselves and onto others (Dodson 1998; Hays, 2003; Pemberton et al. 2016; McCormack 2004; Shildrick and MacDonald 2013); that they appealed to the limits of their responsibility by arguing they had little other choice than to claim welfare (Pemberton et al. 2016); and who condemned the politicians that condemned them (Skeggs and Loveday 2012), it additionally uncovers a line of narrative response pertaining to moral-economic claims for social inclusion and citizenship. Here, participants asserted a reciprocal right to inclusion and thus support from the state because they felt they had contributed to national society in a number of moral-economic ways. Their narratives served to redraw the stigmatic moral boundaries that had been drawn around them and signify they were moral citizens (Lamont 2000; Lamont et al. 2016) who had a social right to receive adequate and non-stigmatised welfare.

Participants made these claims for their inclusion into the citizenry, not through any apparent organised political or legal repertoires, but by appealing to available and valuable moral norms surrounding care and reciprocity. Despite the burning impact of contracts and markets on social life, reciprocity remains a guiding principle to many aspects of human interaction (Gouldner 1960; Mauss 1970 [1954]; Polanyi 2001 [1944]; Tonnies 1931 [1912]). It frames much informal association as well as how individuals relate to larger collectives such as states, and it is one way through which states legitimise taxation (Levi 1997; Williamson 2017). Moreover, amongst the participants, most of whom were, like welfare recipients in general, women, they drew on moral claims to their inclusion through the care work they were involved in. Like reciprocity, care is also an available normative value – and a fundamental one – without which societies could hardly exist (Ferguson 2015). Reciprocal and caring activity thus signalled forms of moral-economic value which participants, in the absence of any organised political scripts, were able to draw on in order to simultaneously signify their morality (Dodson 2007; Lamont 2000) *and* their economy. Such appeals have, however, been smothered by exclusionary boundaries and political and public discourse whereby the moral-economic activity of welfare claimants is rendered silent and invisible. One outcome of this is that the benevolent individuals and institutions that claim to speak on behalf of the poor raise very different kinds of narratives to assert the rights of low-income groups and these resonate little with the moral worlds of the participants in this paper.

## Moral Economies, Markets, and the Boundaries of Citizenship

In E.P. Thompson’s (1971) popularisation of the term moral economy, he suggested that the regular occurrence of bread riots across England in the eighteenth century were not an anomic response to hunger but were instead framed by a normative tradition wedded to long-standing plebeian-patrician relations. In response to the development of market-based price-setting for grain and bread, the poor asserted their right to these necessities at a price that was, regardless of booms and slumps in market prices, affordable to them and which took account of the precariousness of their incomes. James Scott (1976) later developed this concept and applied it to the culture of peasants in twentieth century south-east Asia. Scott found remarkable similarities in the peasant uprisings in Asia as Thompson had uncovered in early capitalist England, outlining two fundamental aspects of moral economy which the peasants’ derived from their patron-client relationships and from their relationships with one another against a backdrop of their substance lives. These were notions of fundamental rights for help from overlords with substance in hard times, and norms of reciprocity. They were inviolable and, if transgressed, led the peasants to revolt.

The term moral economy has since come to take on a broad array of meanings (see Gotz 2015). Indeed, the very existence of welfare provision can be seen to be balanced on the support of widespread public moral-economic reasoning about the necessity for state support (Mau 2003; Williamson 2017). Moreover, UK government rhetoric surrounding twenty-first century economic austerity has presented austerity as a moral action – repeatedly using the phrases ‘fairness,’ ‘responsibility,’ and ‘value’ in order to appeal to citizens to see austerity as morally correct (Clarke and Newman 2012; Morris 2016; Schui 2014). Additionally, as I have mentioned, government justifications for welfare cuts which paint welfare recipients as an irresponsible underclass, too provide an underlying moral-economic logic for welfare reform (Clarke and Newman 2012). Essentially, government rhetoric implies that those without independent incomes are not deserving citizens who thus cannot expect their rights as given.

The UK government’s moral reasoning is also, of course, deeply intertwined with neo-liberal economic reasoning whereby ‘fairness’ – and the whole notion of economic austerity itself – was an ideological moral-economic reaction or adaptation to a financial crisis and economic recession. Within this reasoning, the notion of social citizenship and collective social rights has become increasingly eroded and marketized. This is distinct from Marshall’s (1991 [1950]) observations of post-war citizenship where the UK welfare state provided, at least as an ideal (see Garland 2015), almost universally inclusive benefits for those afflicted by unemployment, caring responsibilities, and ill-health. The post-war welfare system was embedded in a notion of collective social insurance that removed the risks of such afflictions away from individuals and private charities and onto the national collective (see Hills 2015) Yet, as argued by Margret Somers (2008), neo-liberalism more broadly has resurrected a ‘*quid pro quo*’ notion of citizenship based on material inclusion and individualism. Such a contractual and economistic basis of citizenship has eroded the collective foundations of the welfare state – and also forms of moral activity that do not directly produce money capitol – in particular the work of care. Reform has also continued to redraw the lines of responsibility expecting almost all people to be able to support themselves regardless of the broader economy or their personal circumstances.

Of course, neo-liberal morality and its shift towards individual and material citizenship rights is not a completely new feature of political economy, nor one that is completely accepted by all people – alternative moralities will always exist. Yet state domination of moral discourses has tipped the value of broader moralities in particular directions putting more or less emphasis on framing a dominant morality (Hall and Lamont 2013). Consequently, while the narratives of people directly affected by the Welfare Reform Act have gone largely unheard, other moral counter-narratives to UK welfare austerity have arisen from more vocal humanitarian, political and campaign groups. The content of these can be classified into three distinct forms (Morris 2016): ‘economic rationality’ – arguing against the austerity of welfare reform through using data and evidence to question its logic – particularly the degree to which it will save costs; ‘legality’ – appeals to legally established rights to state protection and support for all human beings – particularly for vulnerable groups like women, children and the disabled; and ‘morality’ – absolutist claims about the immorality of human suffering and the necessity for the means of survival which the state must provide.

As I show below, however, these critical narratives resonate little with the claims of welfare recipients themselves. Drawing on a moral economy based around notions of care, reciprocity, and the limits of responsibility, participants made claims to their being moral and economic citizens – and they thus asserted their rights to social protection against the difficult financial circumstances they had found themselves in. Participants had and were engaging in moral-economic activity through the work of care and the previous taxation that they had paid to the national purse, which they repeatedly spoke about in order to raise their credentials as citizens that they felt had been denied to them under welfare reform*.* These claims fit with the notions of social rights that peaked in the UK the mid-twentieth century and they also echo the moral economies of the poor described by both Thompson and Scott. They can be seen as an available moral-economic logic raised to challenge the exclusionary nature of austere welfare, and which provide, at least some, personal resilience to welfare stigma. This logic has, however, been buried under more powerful moral narratives transmitted by both government and their opponents who claim to represent low-income groups.

## Method

The fieldwork underpinning this article was conducted in summer 2014 as part of a ‘Knowledge Transfer Partnership’ between the Essex Housing Officers’ Group (a consortium of social housing providers in Essex – ‘EHOG’) and the University of Essex, funded jointly by EHOG and Innovate UK (grant number: 9309). In the immediate wake of the Welfare Reform Act, EHOG experienced significant growth in non-payment of rents and, later, an ensuing increase in evictions, which had strained their budgets and ability to re-pay mortgages. The project was thus commissioned to better understand the effects of the changing welfare system on social housing tenants and to find potential ways to prevent their rent arrears.

Following two exploratory focus groups with local people, letters were sent, via EHOG, inviting individuals affected by the Act to talk to an interviewer about their experiences, for which they would receive a £10 (approximately $15) supermarket voucher. This yielded 119 agreements from which a smaller number of participants were selected for interview via a framework that generally reflected the broader population of welfare claimants. Selection was also pragmatic as the fieldworker had to travel on public transport, often to quite inaccessible locations, to conduct interviews, while maintaining her safety by, for example, interviewing men only in public locations.

**Fig. 1:**
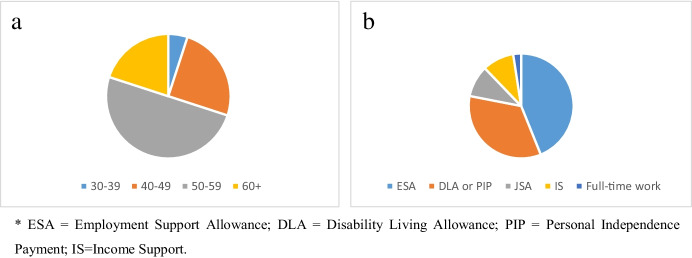
**a** Age range of particpants, **b **Types of benefits recieved by participants*

The fieldworker conducted 36 in-depth interviews either in participants’ homes or local cafes, and the interviews ranged from between 40 to 110 minutes. All interviews were audio-recorded and transcribed by a private company. One interview was later rejected because the respondent was a pensioner who had not been affected by the reform. Of the remaining 35 interviews, 30 were one-to-one interviews, and five were ‘double’ interviews involving two participants (four of these were with married couples and one with a group of two friends). The total number of participants was thus 40. Their age ranges and the type of welfare benefits each received are represented in Fig. 1a and b^2^ below, and fuller details can be found in the Appendix. For reasons of confidentiality, all participant names have been changed.

The distributions of claimant age and benefits type were broadly in line with the national picture of welfare claimants (see DWP 2014), excluding old age pensioners, although as the sample was relatively small and based only in Essex, the findings cannot be effectively generalised to the total population.

All participants had been materially affected by welfare reform through having to pay Council Tax contributions. One was also subject to the Benefit Cap, and another by both the Cap and the Over Occupation Penalty (OOP). The remaining 33 were all subject to the OOP and, of these, only one had moved to a smaller property as a result. All but one participant was classified by the interviewer as white British.

Interview questions were formulated so as to understand the effects of welfare reform on participants’ income and daily lives and to uncover how they managed their shrinking budgets. Based on initial analysis of this data a report was produced for EHOG (Thiel et al. 2015). However, in every interview, participants spoke vociferously about the increased stigma they felt they were subject to under the reform and they offered responses as to why they should not be. This present article thus represents a re-analysis of the interview data with a particular emphasis on drawing-out the participants’ narrative responses to the difficult material and moral situations that they had been placed under – responses that emphasised their moral-economic claims to be considered as full citizens. It begins with a summary of the material effects of welfare reform on the participants and their dominant interpretations of that, before going on to analyse the stigma they felt they were subject to and their critical responses to that.

## Material Effects: Poverty, Precariousness, and Punishment

Primarily as a result of the OOP and obligatory council tax deductions, all participants had found themselves under severe and increased financial strain. Depending on which types of benefits received, their weekly incomes ranged (not including benefits for rent payment) from a maximum of approximately £110 ($165), to a minimum of around £70 ($105). Changes in council tax contributions had meant deductions to these incomes of, on average, five or six pounds per week, and penalties for ‘over occupation’ had cost approximately £14 for each ‘spare’ room per week. Two participants also had to make extra contributions towards their rent payments as a result of the benefit cap.

These penalties and deductions were said to have reduced weekly incomes, after paying utility bills, to as little as £15 or £20 ($23–30) per week from which to buy food and other essentials. The result was that most participants had fallen into spiralling debt to utility and loan companies, with some having to apply to become bankrupt and many others using foodbanks (for detailed descriptions of the financial situations of people affected by UK Welfare Reform see Thiel et al. 2015; Real Life Reform 2013a, b, 2014a, b, c, 2015). Participants consequently said that their everyday lives had become increasingly difficult to manage since the reform and they displayed a series of shared interpretations of their situation. The dominant one was that their lives had been transformed to mere ‘existence’ (see also MacDonald et al. 2014). Mrs. Woods was representative:

Sit in day after day, night after night, just get on with it… It's not living, it's an existence. We just exist, we don't actually live. Does that make sense? Although we breathe, we just exist, we don't live.

‘Existing not living’ indicates a profound sense of exclusion from the kind of lives that participants thought most other people in the UK lived. Due to their material circumstances, participants could not live in the ways that they saw as normal – they were unable to do things other than ‘Sit in day after day, night after night.’ Restricted finances prevented them from engaging in normal leisure activities, buying normal food and clothing, and having normal social engagements. Yet, they could ‘exist’ – their welfare benefits in combination with charities, foodbanks and informal support networks did enable a basic substance but that was not interpreted as normal.

Their sense of exclusion was not solely material but it also resided in the way in which participants experienced the welfare system itself. They said their situation and ensuing treatment was eating away at their individuality – they had become ‘numbers’ in an uncaring and punitive system. Ms. Chester’s words captured these features of life under the reform:

They expect you to live. I exist, I don't live. I exist from week to week… I’ve worked all my life and this is what you get at the end of it - no help - you're just classed as a number and you exist… You can't catch up [with finances] so you're forever in this hamster wheel going round and round and round and round and round, and you hope one day that you might fall off. No other way is there? It's just an existence … I’ve become a non-person, I’ve become a number. I’ve become somebody that doesn't exist apart from on paper that owes this amount and owes that amount. I’m not human anymore.

Under reform, participants had become subject to new and increased tests of conditionality that aimed to ‘activate’ them to become more responsible for themselves and decrease their reliance on benefits. Yet such conditions failed to take into account the complexities of participants’ lives (Hays 2003; Morris 2019), and they were thus interpreted as inflexible and punitive. For instance, although many participants were classified as over-occupying their property, they claimed to either need the ‘spare’ room (commonly for family members or carers to use) or that there were no smaller properties available or, at least, no suitable smaller properties (see Joseph Roundtree Foundation 2014; Thiel et al. 2015). Additionally, most participants with disabilities said that the Work Capability Assessments (administered, at the time, by the private information technology company Atos) they were subject to were too simplistic and rigid to be able to take account of their complex disability but they had nonetheless been re-graded as able to work or attend work training – which, if they did not do, they would be penalised further through their already meagre benefits being cut. These conditions, combined with the material effects of not being able to live a normal life, led participants to feeling de-individuated and stripped of their status as full human beings.

Ms. Ward, who was disabled by a ‘traumatic brain injury,’ provided a narrative about the welfare bureaucracy and illuminated how that had created a sense of insecurity:

They're a task in itself the [claim] forms but, you know, I done them and I will be assessed again next year I think. So it's… a constant threat that all of a sudden you're going to have no money…. Next year I’ll be reassessed again now for this new PIP, Personal Independence Payment… But I mean my disability is never going to get any better… It's like living on a knife edge… it's hanging over you all the time they could just take it away. And [as] we’re living on a budget there isn't any spare, if that money goes you're instantly down if you see what I mean… You're living constantly on edge because everyone's kind of threatening you... I don't know it is a very uneasy… you don't feel safe, you don't feel secure… So I mean you dread every brown envelope that comes through the door.

Participants said that increased testing and conditionality meant they could not reliably know what amount of benefits they would receive from one week or month to the next. This situation was amplified by continual appeals against what they saw as unfair decisions, and a resulting situation of receiving underpayments, overpayments, and delayed payments, making for more insecurity and a difficulty to plan for the future. For instance, most of those affected by the OOP had formally appealed claiming that they needed the extra room or could not reasonably find another suitable dwelling. Many had won these appeals and were awarded Discretionary Housing Allowance to fill the OOP deduction. However, this was awarded only on a discretionary and temporary bases (from three to six months) after which participants had to reapply, and, in the meantime, default on their rent payments. Ms. Dixon provided an example of the effect of the OOP on her sense of security:

These are suppose to be homes that you've got for life because you're not in a position to be able to go out and rent privately… You rely on your social housing as being your main security… and this makes it all very very kind of edgy… Yeah it does cause a lot of… stress because they're basically taking away your basic needs… like security, warmth and food.

The welfare system was interpreted as increasingly insecure and precarious where participants experienced ‘living on a knife edge,’ getting into debt, and being threatened by bailiffs, court summons, and eviction. Despite some creative and skilful approaches to budgeting their meagre incomes (Thiel et al. 2015), managing such insecure and unstable welfare benefits was no easy task. Most participants had fallen into debt, making it increasingly difficult to budget and so on. This provoked considerable stress and strain – both in terms of security of residence and security of income. Those deemed as fit for work would continually have to formally prove their willingness to find employment or have their benefits cut; the sick and disabled could become suddenly and, in their view, unfairly, compelled to find paid work; and those deemed ‘over-occupying’ properties encouraged to move to another residence through considerable deductions to their meagre incomes. Here, the notion of being provided a home by the state was effectively degraded under the reform to being provided a temporary dwelling, and the ensuing sense of insecurity led many participants to envisage only a very dark future.

The regular assessments participants were compelled to undergo put a constant strain on them to find paid work or move to smaller dwellings when, in their minds, they could not do so. The ensuing financial penalties and treatment by the benefits system were therefore interpreted as punitive. Participants’ characterisation of their lives as ‘existence’ was thus frequently accompanied by another interpretation – that rather than being a source of social welfare provided for the needy, welfare had become a source of punishment. Mr. Lane appealed to this interpretation in stark terms:

They don't care if you live or die… I could have died and nobody would have cared. That's how I feel you know, just another number, just another house… It's in comparison with ethnic cleansing. As I say they're punishing those who don't have the money.

Participants’ sense of being punished ties with broader sociological analyses of how market-dominated states have come to manage the poor (Rodger 2008; Wacquant 2009). It can be seen here to be a feature of the reform that models social welfare in an actuarial market-bound framework which supplies only negative sanctions and none or, very few, positive incentives. The single incentive was to find paid employment – which, for many with complex needs and lives intermeshed with the needs of others, seemed impossible (see also Hays 2003). Mr. Holmes summed up this model: ‘They don't incentivise, they bully. It's a case of you take that job or you get no money, that is their attitude.’

These outcomes of reform can be conceptualised as a renewed notion of ‘less eligibility’ whereby just as conditions of employment had been degraded in post-industrial Britain (Standing 2014), so had the conditions of welfare. Reform had created a less inclusive and more precarious type of welfare provision, and this was keenly felt by those in receipt of it. The next section outlines the more symbolic albeit, no less insidious, effects of the stigma associated with reform.

## Symbolic Effects: ‘They're just Greedy Scum’

As a number of studies have illustrated (Clarke and Newman 2012; Jensen and Tyler 2015; Morris 2016; Slater 2012; Wiggan 2012), government statements supporting and legitimising welfare reform paint welfare recipients as trapped in a culture of worklessness, portraying them as actually able to do paid work if only their attitudes were changed (see also Hays 2003; McCormack 2004; Rogers-Dillon 1995). The implication is that the problem of unemployment is a problem of attitude – an attitude of entitlement to welfare for having done nothing to deserve it. Welfare recipients are thus officially framed as a group who take from the nation but do not give anything in return – akin to thieves or scroungers. Reform thus attempts to (negatively) incentivise the ‘right’ attitudes and wheedle out those falsely claiming.

Portraying welfare recipients in this way serves to weaken broader public support for welfare provision (Taylor-Gooby et al. 2019) and thus legitimate the reform but, in doing so, it re-cast a powerful stigma over welfare claimants – and this was something that the participants were acutely sensitive to (see also Dodson 1998; Pemberton et al. 2016). Mr. Wicker spoke for many:

I mean straight away if you're a working person… like my son or my daughter, well, you get their opinion… what they think of that person that's basically sitting on her arse, claiming she's depressed but she's earning money [and] not declaring it you know. Straight away they're going to turn round and say… ‘they're just greedy scum. They're not helping the country, they're not doing anything, they're… just out for their selves’…. And that's the stigma that comes with it now, and this government has made it worse over the last few years.

Participants’ perception of such welfare folk devils or, ‘greedy scum,’ had grown mostly from Government pronouncements, TV programs, and the press, rather than their genuine life experience (see also Shildrick and Macdonald 2013). Their sense of stigma was, moreover, compounded by actual interactions with the local council and job centre staff and the ensuing bureaucracy which formed the various health, living and availability for work assessments (see also Dodson 1998; Hays 2003). Mr. and Mrs. Brown illuminated this issue:

*Mr. Brown*: They say, well there's a discretionary [housing allowance] fund but every six months we have to apply for the discretionary fund to pay the bedroom tax… But they treat you like you're a scrounger you understand…

*Mrs. Brown*: It's like going… cap in hand, like begging, that's what we feel like - that we've got to go and beg for this money because we can't afford to have two bedrooms.

*Mr. Brown*. It’s so degrading

The combination of powerful negative discourses on welfare claimants, an increasingly conditional and bureaucratic system, and perceptions of judgemental welfare staff, led participants to feel like they were treated as begging or scrounging for ‘hand-outs’ or ‘gifts’ from welfare system (see also Boland and Griffin 2016; Wiggan 2012).^3^ This signalled to participants that the state viewed them as having no rights to state support or, that to have a right to continue to receive it, they continually had to prove their moral worth in that they could not reasonably do or find paid work or smaller dwellings. Their perception was that welfare had become akin to private charity that one had to beg for.

The combination of these sources of stigma had reached such a point that some participants said that being disabled in itself had become equated to being a lying scrounger (see also Peacock et al. 2014):

One thing we've noticed over the last… couple of years, if I’m out with [my wife], whereas people use to be like ‘oh she's disabled, we'll get out of her way’... we've had people walk into her… as if she's almost invisible, not quite invisible – you can see her there but she's totally insignificant... [She’s] got no rights anymore because she's obviously disabled, she's obviously not working, she's obviously on benefits, so, ‘we're better than they are.’ (Mr Painter)

Mr. Wicker expressed a similar view:

It's very hard being disabled in this country now, it can be just as bad as being… foreign... People have changed since the Conservatives have got in. I know… they caught a lot of cheats and that's fair enough you know, but because they caught all the cheats, it's in the paper all the time, general public now just think everybody's the same.

Mr. Wicker draws a comparison between his own treatment as a person with disabilities and the foreigner. As I expand on below, ‘foreigners’ were seen as outside of the national moral-economic system and thus non-deserving of citizenship rights and welfare. This indicates how participants like Mr. Wicker experienced and expected to be labelled as outsiders who took from the state but who were not entitled or deserved to do so. Despite this, not one participant saw themselves as a non-deserving scrounger with no entitlement to a fair and secure form of welfare – they were simply treated like that and were consequently angered, stressed, and left feeling punished. Nonetheless, as elaborated in the next section, participants did accept dominant representations of morally bereft and bogus welfare cheats as real and, by doing so, they reproduced and reinforced government and media images of welfare ‘Others.’

### ‘Others’ and the Passing of Stigma

I do think the benefit system needed to be reformed but I just think the way he's [P.M. David Cameron] gone about it, what it's actually done is, it's hurt all the people that actually need the money. And the people that are fiddling it and conning the benefits system are still at it because they know exactly what to do… And it's not affecting them, it's affecting people that actually need the money. (Ms. Paul)

Participants’ views about the aims of welfare reform raised a paradox in that despite the punishment, insecurity, and insidious stigma they experienced, they all accepted one of the main moral tenets underlying reform – that there were some people cheating the system who needed to be screened out (see also Hayes 2003; McCormack 2004). What they did reject and denounce, however, was the *targeting* and *process* of the reform, which they saw as being delivered, through misrecognition of their situations, in the wrong ways and aimed at the wrong people – at people like themselves who were not cheating the system. This section outlines that paradox illustrating, firstly, participants’ views of the reform project and the undeserving skivers the government had outlined in order to legitimate it, before moving to examine how participants redrew moral boundaries in order to differentiate themselves from that.

Although participants had been negatively affected by welfare reform, many also said that they were ‘grateful’ to receive any welfare at all. A number additionally said that the situation they were experiencing was probably better than for other claimants (see also Kissane 2012). For instance, some participants with disabilities reported that they were likely in a better situation than others because they received more finances and they were not subject to such regular assessments. As Ms. Blair said: ‘In a way I’m quite lucky because I’m classed as severely disabled so I get extra benefit. God only knows how people on Job Seekers [Allowance] survive, I don't have a clue.’

It was also common for participants to draw comparisons to other countries that they felt provided less for the poor. Ms. Blair continued:

I’m not a scrounger, but that is what gets to you, and the media have the British people believing that we're all scroungers. We're not all scroungers, we're not. We need help, we need support, that's all. And thank God I live in Britain because that's what Britain's all about. I mean I could be living somewhere else in the world and sitting on the streets.

Feeling grateful for any welfare provision at all and fixing the UK as a nation unlike others that did supply at least some level of subsistence living for the needy was a source of some sanctification for the UK nation and its welfare. Yet, in raising welfare to this level, those others that were seen to defraud the system were severely demonised. In this way, participant narratives about welfare cheats were almost indistinguishable from those of government and popular media. Consequently, despite the views that some participants felt themselves better off and luckier than some others, this was not their dominant view of ‘Other’ welfare recipients. Many other claimants were instead seen as successfully playing and cheating the welfare system, and the system was seen to play back to them.

Such morally bereft others were the ‘usual suspects’ of marginal British society: irresponsible young mothers; undeserving migrants; and youth locked in a dependency culture who felt entitled to benefits – demons that are all long-standing and reoccurring in the UK (see Cohen 2002). Yet these old ideas about intergenerational dependency and irresponsibility (see Morris 1994; Pearson 1983) had been renewed and amplified under reform, and were, paradoxically, endorsed by welfare claimants themselves. Mr. Copse provided another example:

I think there are people on benefits that are cheating the system. I always thought that if you look at Joe Blogs [who] has got 11 kids and is living off £1,000s worth of benefit and don't work… I don't know how he does it but it's like they just get the wife or girlfriend pregnant, more money to them, which is not the right way. And some of these people… they can work, they're just bone idle, they can't get off their backsides to do it…. You can't… run a country on people sitting down… So all these people just think… ‘oh dad's sitting on his arse all day, I can sit on my arse all day’ – and that is how some families look at it. They're getting so much, so many benefits… I’m not saying that this is every family, it's not, but you read in the papers where they've got 11 kids and they're not working.

Participants felt that morally bereft others were able to play the system and reap its rewards – ‘getting so much, so many benefits.’ Although less common, some participants also directed their moral outrage to another stable folk devil – the migrant,^4^ who was also seen as able to effectively exploit the welfare system**.** A**s** Mrs. Davis told us:

Everybody's up in arms with all this immigrants thing at the moment as well aren't they? The people here that need the help don't get it or they get it by abusing the system, and then all these people come over from other countries, wiggle the system and get what they want.

Participants were, just like the enduring public and political debates about welfare in general (Morris 1994), drawing lines and boundaries about who was a deserving citizen or not. Akin to government statements, they saw the non-deserving as those that claimed to not be able to work when in reality they could, or as those who had not contributed anything into the national moral-economic purse and thus did not deserve to take anything out. Mrs. Squire clearly expressed this latter – reciprocal – notion of what deserving of benefits meant to her:

Sometimes when you see single parents that are working and doing their best to bring up a child not on welfare – and there are a lot of them – and then you see other ones whose parents have been on welfare and they're on welfare, and the children are being brought up on welfare. It's quite difficult knowing that they've never worked and they're taking money out of the system before they've put in any.

Not ‘paying-in’ or ‘putting-in’ meant more to participants than simply monetary or material inputs. As I elaborate below, it also included non-remunerated and moral contributions to the national ‘purse’ in terms of being good citizens – especially those moral activities related to caring, community and voluntary work. These conceptualisations of simultaneous moral and material input were framed by norms of reciprocity that pattern much everyday social activity (Gouldner 1960; Mauss, 1970 [1954]; Polanyi 2001 [1944]; Thiel 2010, 2012) whereby if one did not contribute, they were seen to have no reciprocal right to draw welfare.

## Reciprocal Citizens and Social Citizenship

Through condemning those who failed to reciprocate – who took without giving – participants contrasted themselves as those who had paid-in and thus had a moral right to adequate support. One consequence was that participants felt welfare reform had been targeted incorrectly – punishing themselves who had contributed and were in real need – rather than the apparently ubiquitous scrounger, underserving immigrant, or irresponsible mother. These views presented both a continuation of the demonisation of welfare claimants but also a partial counter-narrative and some personal resilience to the effects of reform and its stigmatic message. It was here where one conflict of perspectives emerged between participants and the dominant governmental discourse – over the *value* of particular moral-economic inputs.

For many, especially the majority of participants that had been in paid work for most of their lives but had later lost their job, become too disabled to work, or had taken on the care of a disabled family member, one primary input they ‘deposited’ into the national system was their tax and National Insurance when they were in paid employment. Nearly all participants highlighted this so as to underline their right to draw benefits, and, thus, that welfare, for them, was not a charitable gift but an entitlement. Ms. Blair provided an example:

I do believe in the old saying ‘charity starts at home’ but it isn't charity because we've paid into it – we've paid into a National Insurance system. When I was working I paid National Insurance – the clue is in the name – *insurance*! You pay insurance premiums, when things go wrong, they pay up. That might be quite simple but that's the way I look at.

Ms. Blair’s statement draws on a sense of fairness parallel to the notions of social citizenship associated with the post-war UK welfare state which was designed around the idea of collective insurance. Here, its architect, William Beveridge, was aware of the need to present support as reciprocal so as to avoid the stigma of it being seen as an un-earned charitable gift that people had to beg for (see, for example, Hills 2015). Beveridge’s social insurance system thus operated as a collective social entitlement that was paid-for and, unlike a gift, it would have few moral strings attached – and, thereby, in theory at least, be free of strict conditions. The system aimed to protect against the costs of unemployment and sickness as, for example, becoming physically frail in later life was something that Beveridge and his public saw could happen to anyone – rich or poor – although, of course, unemployment and sickness are much more likely for manual workers, and, in turn, more likely to prevent such workers from continuing to do paid work.

As Marshall (1991 [1950]) argues, the post-war welfare state emerged out of considerable struggles for equal social citizenship – struggles that were bolstered by an expectation that the state would reciprocate its citizens for their life and labour in the two world wars. However, despite the symbolic resonance of the name national *insurance*, neo-liberal morality has eaten away at these collective ideals, pitting citizenship as an individualised quid pro quo system (Somers 2008) blind to the pitfalls and complexities of people’s lives – and any sustained struggle against that has been partial at best. Moreover, in moving from its post-war collective insurance system to a more prescriptive and precarious form, the long-standing stigmatic images of welfare claimants have been amplified, and this stigma acts to wash away the value of welfare recipients’ moral-economic inputs and, ultimately, their value as human beings.

These developments have pushed the status of welfare claimants almost to the symbolic position of the (liberal) Victorian poor deemed only good enough for charity. Participants, however, expressed incredulity at this lack of recognition. Ms. Blair continued:

I worked as long as I could, paid my taxes, paid my National Insurance, paid my rent. I brought up two children on my own because I was divorced and worked for a living… I had polio as a child and now I’ve got what they call post-polio which brings on fatigue and pain and muscle weakness... I’m not looking for sympathy – that's what I live with – but what it means is I can no longer work… But I worked as long as I could, so I do believe I’ve paid into the system. I’ve paid my dues, now I need help. Is it so bad to ask for help?

Alluding to her moral-economic inputs of tax, National Insurance and child rearing, Ms. Blair also makes appeals to the simple fact that she had worked – implying that she was not work-shy. Such sentiment could be seen in many of the interviews where the value of work was expressed over and over: participants were moral beings who had put effort, good intention, and money into the national purse when they could possibly do so. Paid work was also spoken about as a ‘good’ in itself – quite separate to its financial benefits (see, for example, Ms. Grace and Ms. Jackson below), and participants regularly expressed how their parents and children were also ‘hard workers’ – drawing a form of intergenerational morality over themselves. To restate the obvious – but an obviousness continually denied – the participants were not morally bereft: they appealed to fundamental moral notions – but notions that had been flattened by a dominant discourse.

Such views represented a form of moral-economic reasoning that underpinned participants’ ideas of social citizenship in which they asserted their own rights to welfare and symbolically withdrew the rights of others. These basic notions of reciprocity extended only nationally and they denied any basic or God-given human or sacred legal right to state support. As a consequence, ‘immigrants,’ for instance, were outside of participants’ notions about who deserved to receive UK welfare, as were those Others that seen to not morally contribute anything.

### Care and Responsibility

Participants like Ms. Blair said they had become too sick to continue paid employment, and many others had stopped their employment to care for sick children or loved ones. Here, a moral narrative about responsibility and its limits emerged in participants’ talk whereby they claimed to not be responsible for their loved ones becoming sick but were responsible to care for them since this had occurred. Alongside this, they made appeals to recognise the informal and, largely, non-remunerated, care work that they had contributed to the national moral-economic purse but which went unrecognised. Mrs. Squire provided an example:

I have paid my taxes, I have contributed to society in various ways and now you're punished for doing the right thing, for looking after your husband – that's what it feels like… He [my husband] was a double above the knee amputee. It involved a lot of caring and a lot of physical work… Maybe I should have just stayed at work and let somebody else look after him and then I wouldn't be in this situation now... Nobody likes claiming money from the government but when you've paid-in expecting to get help when you need it, to then be punished because of circumstances beyond your control… It was the right decision, I couldn't morally accept somebody else looking after him… I mean financially it would be stupid to give up work to look after him.

Mrs. Squire had taken a moral decision to care for her husband whose disability was outside of her control but, in doing so, she had been punished for ‘doing the right thing’. Part of this narrative echoes observations about how women, in particular, can be torn by conflicting values of caring versus paid-work (Hays 2003; Hennessy 2009a; Morris 1994). On this latter theme, Mrs. Cotton demonstrated the moral dilemma that the welfare system had put her under as the mother of a child with severe disabilities:

I kept thinking about going back [to work], and I thought, do you know what, I can't do it. I just couldn't do it. I couldn't leave him [my disabled son] really. I just thought at the end of the day he's like me flesh and blood and I’d never forgive myself if something happened to him and I was out there working you know... But he's had some pretty, well, quite a few times, where he's nearly died, and I just think what's more important? So to a certain extent I would rather struggle and support him than I would work.

The ethic of care – a rather uncontroversial appeal to morality – despite being raised regularly by participants, was something hidden in dominant UK discourses about welfare provision and the ‘economy’ more broadly. In the words of Ms. Cotton, she had taken a moral, over a financial, decision to care for her son but, by doing so, she had become impoverished, stigmatised, and punished.

Claims for the moral value of care by people on low-incomes is not uncommon in the qualitative literature (particularly from the US – see Dodson 1998, 2007; Hennessy 2009a, b; Lamont 2000; McCormack 2004), which sees them regularly raise the value of care as a counterpoint to the more dominant values of paid work and materialism. The morality of care is an available value and, like reciprocity, it is probably more central to lives of low-income groups where informal networks and care and support is, as I have said, a major part of how they manage to exist on low incomes (cf. Lamont 2000). The value of care was also, like norms of reciprocity, drawn from the norms and rules that framed the everyday lives of participants – and not from any apparent organised political or legal discourse. It can be seen as a moral precept with the ability to be extended from the part it plays in everyday interpersonal life, out to the organization of entire nations (see Skocpol 1995). Just as individual people cannot adequately exist without being nurtured and cared for – neither can collectives (Ferguson 2015). Despite this, care continues to be underrecognized as a form of work and, in neo-liberalism, is deemed something that should be supported only by waged work (Dodson 1998). Care supported by the state has come to be cast as invisible at best and immoral at worst.

The invisibility of care work has, of course, a history much longer than welfare reform and neo-liberalism. It is a long-standing form of patriarchal misrecognition of a type of labor (Finch and Groves 1983; Oakley 1974; Ungerson 1987) which continues to affect women’s access to full citizenship (see, for example, Lister 1995, 2007). It has also been demonstrated that the value of care work is not, however, invisible in all contemporary societies – and it could thus be otherwise (Lyon and Glucksmann 2008). Nonetheless, parts of the rhetoric surrounding UK Welfare Reform have strengthened this invisibility – stalemating decades of feminist campaigns and indicating how UK market capitalism continues to be fundamentally patriarchal. Yet, paradoxically, gendered norms about, particularly women’s role as carers, remain entrenched. Under reform, carers were thus placed in a moral catch-22 in that woman who chose to do paid work rather than care might experience just as much stigma as those who chose to care rather than do paid work. This was a source of strain for many – men and women – who had to decide the most appropriate course of action and negotiate a moral pathway of largely only negative outcomes.

The value of care was emphasised in particular by participants who had worked most of their lives as unpaid carers and thus had not paid large amounts of tax into the national purse but, instead, elected to care for their young and, sometimes, disabled children. As outlined above, irresponsible mothers were a dominant demon. Consequently, for participants who were mothers and who had not been in paid employment for the majority of their lives, their over-riding symbolic response to reform was framed largely by appeals to having ‘paid-in’ to the national moral-economic purse through caring activities for which they claimed they were morally responsible. Ms. Hill provided an example:

I mean once upon a time it was like frowned upon for a woman to go to work wasn't it? You stayed at home, you cooked the dinner, you collected the kids from school. But now I think that there's this push that everyone should be out at work… I don’t necessarily agree with that and the awful assumption as well that people in my situation are just having babies… I was married to my middle daughter's dad and we didn't receive any benefits at all but then he left me, he divorced, so I then was put in that situation where I was receiving benefits you know... Ideally that's not… what I set out to do … I think the media nowadays and the way they… put across that all single parents are… just having babies… we're all just out to take money from the government… I know there are people out there that are like that… they just want a free ride… but there is a vast majority of people that, you know, that's not their mind-set and I think it's sad… It's just unfair that we've all kind of been bracketed as… being scroungers.

Ms. Hill argues that she ‘should be entitled to look after [her] children because… it wasn't [her] choice to be in that situation.’ She was not responsible for her situation and appealed to moral notions about motherhood. Ms. Grace and Ms. Jackson echoed much of this:

*Ms. Jackson*: Throw me to one side… because you married wrong, because this and that and the way life threw at me… Not what I went out and did deliberately what life threw at me.

*Ms. Grace*: No that's right, I didn't ask for a disabled child.

*Ms. Jackson*: I'm lucky to have come through it.

*Ms. Grace*: If I hadn't had a disabled child I would have had a different life but I didn't ask for a brain damaged child, lack of oxygen at birth… When she was here that was it, my life was over you know what I mean. You had to concentrate on them… I worked when I was young obviously but as soon as I had a disabled child, you know, mentally and physically quite disabled, what do I do? You can't leave them with someone and go to work, you become their carer… If I hadn't have had a disabled child I probably would have got a...

*Ms. Jackson*: Carried on working.

*Ms. Grace*: Carried on working and work me way up and I’d have had a nice...

*Ms. Jackson*: Grace couldn't leave her… So that was her life…

*Ms. Grace*: I would’ve loved to have a career…. Mentally and physically it does you good to go to work but, some people, it just don't happen because of the circumstances – you have a disabled child, that's it, you're knackered… So that was my reason why I couldn't work and… when I did work I’d always been a good worker… But they don't take into account, I mean, I know there's some people that don't work and they don't intend to work and there's nothing wrong with them, and I do understand they don't deserve, you know, keep their house filthy, but they don't look at individual cases you know.

As well as referring to her lack of choice as a result of giving birth to a disabled child, Ms. Grace also alludes to the morality of dirt – an older and class-bound notion of moral respectability (see Thiel 2007) which Ms. Jackson and her frequently spoke about in order to distinguish themselves from welfare ‘others’ (see also Peacock et al. 2014; Tyler 2008). These calls to normative, moral and, perhaps, peculiarly class-bound norms about cleanliness and traditional motherhood highlight another clash of perspectives between participants and governmental logic. While government may have taken social change by the reigns and denied the gendered nature of care, and housing inspectors no longer particularly concerned with sparkling front doorsteps (see Young and Willmott 1957), moral norms about care and cleanliness continued to be integral to participants’ moral worlds. These are forms of morality that are likely particularly valuable for those living at the nadir of the class structure experiencing the brunt end of powerful discourses about ‘scum’ and respectability.

### Responsible Job Seeking

In addition to highlighting the moral-economic inputs they had contributed to the collective national purse, participants simultaneously claimed that conditions outside of their control had led them to needing welfare. They had ‘no choice’ over, for example, their partner leaving them, their child being born disabled, or their becoming disabled. On the other hand, if some participants did have an element of choice, this was pitched as between two almost equally morally suspect choices – working and not caring or caring and not working – a moral catch-22. Moreover, there was not a single participant who pointed *only* to their lack of choice, but such claims were always accompanied by appeals to also having paid-in in some way – their moral-economic narratives were always double-edged.

This was not only the case for the majority of participants who had become carers or too disabled to do paid work, but also for those in receipt of Job Seekers Allowance (JSA): people classed by Government as able bodied and not caring for another – the very demons of government discourse able do paid work if only they were presented with appropriate incentives. However, participants in receipt of JSA claimed that they were moral citizens contributing their effort *until* conditions outside of their control had stopped them from doing so. Indeed, not a single JSA participant had never engaged in paid work, and many had initially stopped paid work due to ill-health or caring responsibilities. Yet, when their health was deemed to have improved enough to find paid work, or their caring responsibilities had ended, they were placed onto JSA and made to continually to prove their willingness for paid work. Ms. Eves, for example, stated above that she had stopped paid work to care for her disabled husband. When he had later died, however, she was transferred from Carers Allowance onto JSA and simultaneously became deemed as over-occupying her property. She said:

My husband was ill and he's now died and the council say downsize, [but] this is my home so it is quite difficult sometimes. And yes you get put on Job Seekers Allowance because there's no widow's pension as such now and I’m of working age. But at 56 you go into the job market and they say ‘what have you been doing for the last seven years - caring for your dying husband?’… I manage the best I can and I go volunteering, so I volunteer along at the hospice collection centre and I volunteer up at the Learning Shop, so I get to meet lots of different people but still no work…. You're out there, you're looking, you send hundreds of CV’s off and you don't get a reply… And I do feel angry about it, I do feel angry. It's not as if I haven't been working, I have paid my taxes, I have contributed to society in various ways.

It must be restated that the proportion of UK welfare recipients who have never been in paid work or those unemployed for long periods of their lives is massively dwarfed by the sick, carers of children and the sick, and the numbers of short-term unemployed. In terms of the latter, as studies have demonstrated (Hills 2015; MacDonald and Marsh 2005; MacDonald et al. 2014) there are only tiny numbers of long-term unemployed in the UK, miniscule by comparison to the number of short-term unemployed who tend to bounce back and forth between unemployment and low paid, short-term jobs (Shildrick et al. 2012; see also Dodson 1998). The dominant public demon pertaining to the morally bereft welfare recipient who takes but does not give anything in return, repeated by the participants many times above, is, in reality, an extreme rarity. The vast majority of people receiving welfare have paid something – morally and financially – into the public purse, and they thus expect to be treated with respect, fairness, and a basic standard of living. Yet, as I have discussed, dominant UK discourse smoothers and silences these observations, serving to exclude welfare claimants from the broader national moral universe.

The next and final empirical section outlines another participant response to the message and process of the reform. Although not mentioned by all participants, this response involved re-drawing moral boundaries through condemning the politicians at the helm of the reform by pointing to their elite form of exclusion from ‘normal’ people and their ensuing *lack* of moral-economic activity.

## The Boundaries of Citizenship

I mean they go in saying ‘the big society’^5^ and all this lot, ‘we're all in this together.’ No, we're not, you're not, it's them and us! I mean the politicians they're fine, they got their money, they got their jobs. They don't live in the real world and yet they expect us to sort of live to their standards, and you can't… (Mr. Holmes)

Although narratives that appealed to notions of responsibility and reciprocity meant that aspects of participants’ responses to reform chimed with some of the moral logic raised by the austerity government, around a third of participants did express more pointed politicised narratives. Similar to participants’ other forms of riposte to reform, however, these responses did not challenge the legitimacy of reform per se but, instead, they contested the legitimacy of those that had ordered the reform, drawing clear boundaries between themselves and the government. In this respect, Mr. and Mrs. Painter’s talk about this and their own perceived class position was revealing:

*Mr. Painter*: It seems to me that we're made to feel a lesser class, a no class.

*Mrs. Painter*: We've gone below working class haven’t we?

*Mr. Painter*: Yeah, yeah, we are no class you know, and while these people that sit in Westminster… Did you know that one per cent of the British population is millionaires… [but] 71 per cent of MPs are millionaires? And they're suppose to represent us? They don't know. They've been to Eton [an exclusive private school].

*Mrs. Painter*: They haven't got a clue have they?

*Mr. Painter*: They've been to private school, they've been to Eton, they've been to Cambridge… they've not had lives, they've not worked you know. If Ian Duncan-Smith hadn't married a rich woman he'd be in this kind of boat…. and he seems to be able to think ‘right I’ll take it out on those...’

*Mrs. Painter*: That can't fight back.

*Mr. painter*: That can't fight back... The poor, the vulnerable, the unemployed… To me it's just ridiculous – these MPs that have got their second homes and their expenses and everything... These MPs are privileged, telling the poor and vulnerable that ‘you're too well off…we've got to take more money off of you.’ It's just a load of bullshit, it really is, and they're living in their mansions, they're… claiming [expenses] for underwear and breakfasts and drinking their cheap champagne in the House of Commons bar.

Mr. and Ms. Painter allude to social class both directly and indirectly, categorising themselves rather in the image of the politicians they bemoan as being ‘no class’. Yet they also challenge the legitimacy of those politicians by implying that government is staffed by a wealthy political elite who can know nothing of what it is to be poor. This notion can be seen as embedded in their claims for social citizenship whereby the political class were not included in the imagined collective because they represented a separate group who did not need to work or care – and thus did not contribute to the moral-economic purse. The political class was perceived as providing input only within that separate class – not into the collective purse. Duncan-Smith, for example, was seen as receiving his wealth through marriage not effort, and (below) David Cameron’s wealth as generated through inheritance.

In this way, some participants were re-drawing moral boundaries and devaluing the rhetoric surrounding reform by ‘condemning their condemners’ (Matza and Sykes 1957) who stood outside of the collective world of everyday (moral) citizens – and who thus held little legitimacy from which to penalize (the wrong type of) poor. These claims framed who was and who wasn’t a deserving citizen – with the government deemed not much different from the welfare folk devils who took and gave little in return. Mrs. Chester expressed a similar sentiment:

He's [David Cameron] alright, he's got millions in the bank…. They don't know how… the normal person lives… They really need to come off their high horses and come and look how normal people live because they're not normal people you know. They come from rich families that have got money passed down from generation to generation, [and] live in million-pound houses.

Although such narratives acted to exclude from the moral universe and thus partially de-legitimise the moral claims of government and raise the participants’ status, like the other counter-narratives described above, they centred not on the aims of reform being incorrect but, instead, on the assumption that government did not themselves contribute any moral economic effort and could not understand the situation and effort of everyday people. These, perhaps, class-bound narratives, however, further reveal the inherent reciprocal and social nature of participants’ moral-economic reasoning and its relationship to their notions of social citizenship.

## Conclusion: The Values of Moral Economies

Participants’ dominant interpretation of their experience of welfare reform was that their lives had materially been reduced to a point where they felt excluded from what they saw as normal life and they were instead subject to a subsistence ‘existence.’ Participants were also morally excluded by dominant discourse surrounding welfare reform which stigmatised them as thieves or beggars not deserving of collective support. In opposition to this – what they saw as punishment for circumstances outside of their control – participants drew on moral-economic notions related to reciprocity, care, and the limits of responsibility in order to redraw dominant boundaries and thus assert their right to adequate support from the state. They claimed to be deserving citizens because they had contributed to the national social system through various moral-economic effort until circumstances outside of their control meant they could no longer do so.

At the risk of repeating countless past research studies (for example, Dodson 1998; Hays, 2003; Hennessy 2009a; Hills 2015; MacDonald and Marsh 2005; MacDonald et al. 2014; McCormack, 2004; Morris 1994; Pemberton et al. 2016; Shildrick et al. 2012), participant narratives indicate no evidence of a culture or subculture of welfare, worklessness or entitlement. If they were part of such a culture, they might claim an inalienable right to receive welfare, which none did. Indeed, most participants’ felt grateful to receive benefits. Where they were ungrateful, was the way in which they were targeted by the negative incentives and stigma associated with the reform. They agreed that some people were out to play the welfare system, but not themselves: they had paid-in and they had not chosen to be in receipt of welfare.

The enduring stigma pertaining to the poor and social welfare, and its revitalization under the reform, had however publicly eroded the participants’ value as citizens and rendered their paid work, and unpaid care and community work, as morally and economically invisible. The moral-economic claims made by the participants were thus smothered under the much more dominant discourse that paints them as irresponsible cheats. Their narratives have also been silenced simply because welfare recipients are rarely asked their view or, if they are, these views are treated as unsophisticated and infected with dominant ideology. Yet, what can be seen in the participants’ narratives is a form of moral-economic reasoning bound to notions of social citizenship (Marshall 1991 [1950]) that have, in other ways, become undone by market ideology (Somers 2008). However, this reasoning is also indistinguishable from the pre-industrial forms of moral economy described by Scott (1976) and Thompson (1971) where the poor called for their social rights to adequate assistance from their overlords during times of want.

Participants’ moral-economic reasoning thus reflects what Scott (1976) saw as a call for *social* rights rather than political or legal rights – predominately rights for the return of effort. This is a notion where citizens are those who are made so by their moral and economic contributions to the symbolic community – and that community is then expected to reciprocate in times of need – regardless of fluctuations in economic markets, wars, or government budgets. It is embedded in the moral logic of collective reciprocation rather than one tied to current governmental logic linked into the movements of an abstract economic market and a quid pro quo notion of citizenship.^6^ This moral-economic reasoning was available to pre-industrial peoples just as it is available to contemporary UK welfare claimants because it represents a call for social rights in the absence of more organised or politicised collective repertories to draw from (cf. Lamont 2000), where the reciprocal rules of everyday life are generalised to the imagined collective.

While an aspect of these claims – highlighting the value of informal labor – *has* been a major focus of academic feminism and parts of economic sociology (see, for example, Dowling 2016; Finch and Groves 1983; Glucksmann 1995; Oakley 1974; Ungerson 1987), its intertwine with a moral economy of low-income groups is under-developed. Indeed, in terms of criticisms of welfare reform and austerity, participants’ moral-economy represents a line of moral logic that has been ignored by the more vocal criticisms made by the left, which have predominately called for legal or God-given human rights to adequate welfare (see Morris 2016). Here, then, those subject to the worst effects of marketisation of social relations and their political representatives are talking different languages.

Moreover, critics of austerity appear to see citizenship as a given and not an issue to be raised. What can be seen in this article, however, is that the participants made claims primarily for their inclusion as citizens – political and legal rights were distant, abstract and suspicious figures. As Somers (2008), drawing on Arendt (1979 [1951]), observes, people must first receive social rights in order to achieve legal and political rights (see also Ferguson, 2015). In this context, the bourgeoning moral and social exclusion of welfare claimants – who are predominately women – has serious ramifications. Additionally, participants’ notion of social citizenship was a collective one based around moral-economic practice: something done rather than a static and immutable right. Their inherent notion of social rights thus differs considerably from fixed religious or legal notions of human rights and also from economic-based criticisms of or, support for, austere welfare. It represents a largely hidden way of thinking about the world that the political representatives of low-income groups might hear and build upon.

## Appendix: Respondent details

**Table:**

**Name**	**Age**	**Area of residence in Essex**	**Main benefit(s) received**
Ms. Grace	59	Epping	Employment Support Allowance (ESA)
Ms. Jackson	59	Epping	ESA
Ms. Standage	61	Mersea	Disability Living Allowance (DLA) - low rate.
Ms. Hurren	50s	Colchester	ESA + DLA
Ms. Hill	40s	Colchester	Lone parent benefits
Ms. Potter	34	Colchester	DLA and Job Seekers Allowance (JSA)
Mr. Smith	57	Epping	JSA
Mr. Brown	60s	Epping	ESA
Mr. and Mrs. Woods	Ages not known	Tendring	ESA + DLA (low rate), and Carers Allowance
Mr. and Mrs. Painter	40s	Tendring	DLA and Carers Allowance
Mr. and Mrs. Brown	60s	Tendring	Personal Independence Payment (PIP) and Carers Allowance
Mr. and Mrs. Wicker	50s	Epping	ESA
Mr. Osbourne	37	Tendring	Income support (but signed off as sick)
Mr. Copse	47	Maldon	ESA
Mr. Lane	50s	Colchester	ESA
Mr. Addo	60s	Tendring	JSA
Mrs. Chester	60s	Tendring	ESA
Mrs. Jones	51	Tendring	DLA
Mrs. Sharp	50s	Chelmsford	ESA
Ms. Ward	57	Tendring	ESA and PIP
Mrs. Box	60	Manningtree	Housing Benefit only
Mrs. Squire	50s	Colchester	JSA
Mr. Holmes	40s	Colchester	JSA
Mrs. Beale	48	Tendring	ESA
Mrs. Cotton	50s	Tendring	DLA and Carers Allowance
Mr. Phillips	40s	Colchester	Carers Allowance
Mr. Young	50s	Epping	ESA
Mrs. Davis	40s	Colchester	ESA
Mrs. Lowe	40s	Colchester	Working and receiving Housing Benefit only
Ms. Ulrich	59	Colchester	ESA
Ms. Paul	60	Colchester	DLA and Income Support
Ms. Johnston	50s	Colchester	ESA
Mrs. Hake	40s	Colchester	ESA + PIP
Ms. Dixon	50s	Colchester	ESA
Mrs. Todd	50s	Colchester	ESA
Ms. Blair	59	Manningtree	ESA + DLA

## Footnotes

1 In the run up to the 2019 UK general election the Conservative government announced that the austerity-related freeze on benefit payments in relation to inflation would cease – everything else associated with the Welfare Reform Act would continue. However, in the wake of the COVID 19 pandemic and a sudden increase in unemployment, government awarded a £20 weekly increase to Universal Credit payments. This increase was rescinded in October 2021.

2 This figure is illustrative but not totally accurate as some respondents were unsure of the name of the benefit they received, and others received more than one form of benefit.

3 This aspect of welfare has a longer history than the 2012 Reform Act. For example, it was noted by Bottomore in 1991 that welfare was becoming framed as charity along with rising neo-liberalism.

4 These aspects of their narratives may have been played down due the interviewer being from South America – and whose accent was not native to the UK.

5 The ‘Big Society’ was a concept central to the 2010 Conservative Party Manifesto and regularly mentioned by David Cameron. Its signified increased autonomy to local communities and individual citizens who could better care for and govern their local areas, often voluntarily, without interference or funding from a big central state.

6 In the history of human relations, contractual and quid pro quo exchange is a relatively recent add-on to community interaction and is not the natural state of things (Polyani, 2001 [1944]; Sommers, 2008). Indeed, Polanyi (2001 [1944]) convincingly illustrates that economic markets are always accompanied by various social ‘embedding’ mechanisms developed in order to protect communities from the ill-effects of economic markets. A related argument is made by Garland (2016), drawing on Durkheim, who shows that welfare always exists in all societies in some form or another. In this way, it could be argued that the moral economy raised by participants in this article is a necessary and inevitable cultural embedding mechanism that under-girds human exchange and social welfare (cf. Thiel 2010).
